# Supplementary material for: Hydrolytic Degradability, Cell Tolerance and On-Demand Antibacterial Effect of Electrospun Photodynamically Active Fibres
Source: Pharmaceutics. 2020 Jul 29;12(8):711. doi: 10.3390/pharmaceutics12080711 (PMC7465204; doi:10.3390/pharmaceutics12080711)
Supplement: Supplementary file 1 [file pharmaceutics-12-00711-s001.pdf]

pharmaceutics

Supplementary Materials: Hydrolytic Degradability, Cell Tolerance and On-Demand Antibacterial Effect of Electrospun Photodynamically Active Fibres

Amy Contreras, Michael J. Raxworthy, Simon Wood and Giuseppe Tronci *

Light source and intensity measurements

A 6000-lumen work light (50W, 135 lumen/W, 2800-3200 warm light) was selected as a light source model. A hand-held optical meter (ILT2400, International Light Technologies) was used with a laser line filter (ThorLabs, Inc.) centred at 670±2 nm to determine the light intensity in the spectral regions of MB peak absorbance. The light intensity (mW·cm^{-2}) was measured with and without the filter in 9 distinct locations of the lamp and values averaged. The measurement was repeated three times.

Encpasulation efficiency of PAFs

Discs (Ø 1 cm) of PAFs were individually weighed on an analytical balance prior to 48-incubation in HFIP (5 ml) to enable complete sample dissolution. A standard calibration curve was built via UV-Vis spectrophotometry using MB solutions in HFIP covering the range of MB concentration expected in PAFs. The encapsulation efficiency (EE) of MB in PAFs was calculated according to Equation S1:

$$EE = \frac{m_d}{m_e} \times 100, \qquad \text{Equation (S1)}$$

whereby m_d and m_e are the determined and expected weights of MB in the electrospun samples, respectively.

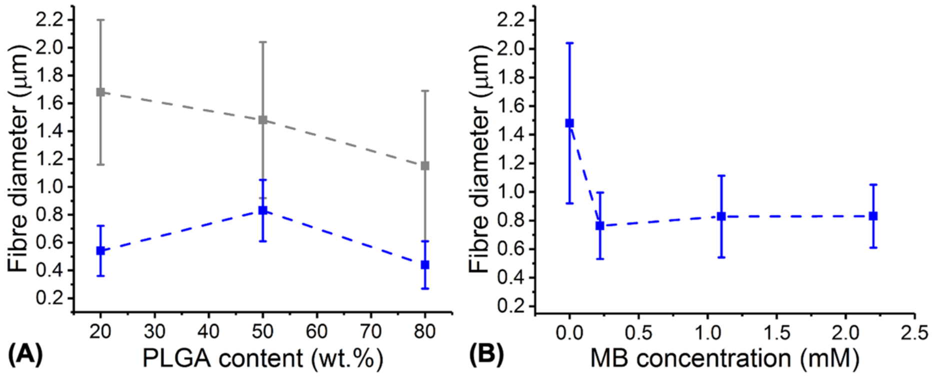

Figure S1. (A): Variation of fibre diameter in PAFs (■) and MB-free fibre controls (■) made from electrospinning solutions containing varied PLGA content and constant MB concentration (2.2 mM). (B) Variation of fibre diameter in PAFs made from electrospinning solutions containing constant PLGA content (50 wt.%) and varied MB concentration.

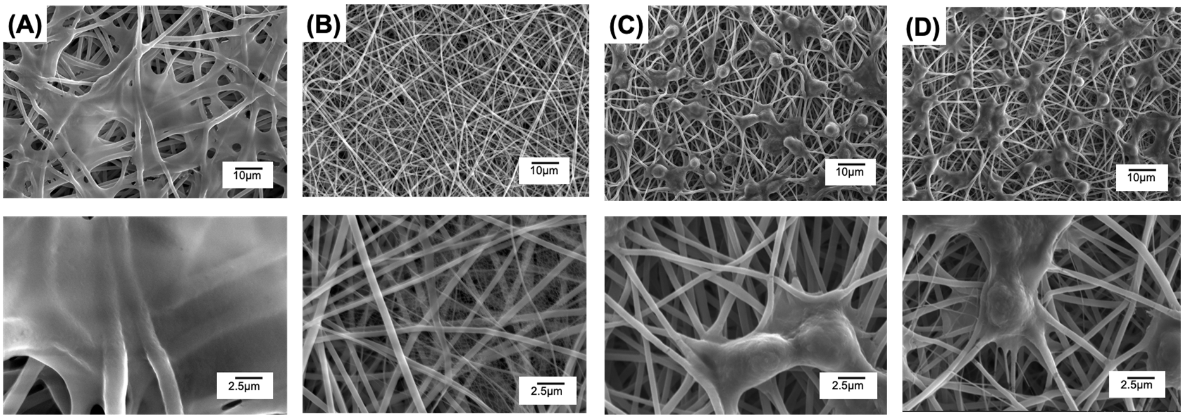

Figure S2. SEM of electrospun fibres cultured with L929 cells for 24 hours. Prior to 24-hour culture, L929 cells were seeded on cell culture medium-equilibrated fibres and exposed to 60-min light irradiation in dark. (A): PLGA50-CL50; (B): MB20-PLGA50-CL50; (C): MB10-PLGA50-CL50; (D): MB2- PLGA50-CL50.

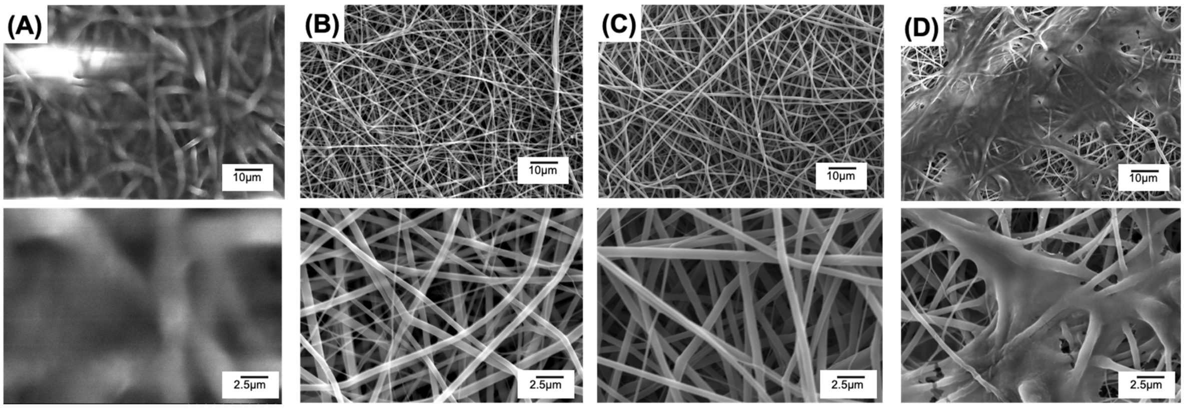

Figure S3. SEM of electrospun fibres cultured with L929 cells for 7 days. Prior to 7-day culture, L929 cells were seeded on cell culture medium-equilibrated fibres and exposed to 60-min light irradiation in dark. (A): PLGA50-CL50; (B): MB20-PLGA50-CL50; (C): MB10-PLGA50-CL50; (D): MB2-PLGA50-CL50.
